# Ideals and primitive elements of some relatively free Lie algebras

**DOI:** 10.1186/s40064-016-2545-2

**Published:** 2016-06-22

**Authors:** Naime Ekici, Zerrin Esmerligil, Dilek Ersalan

**Affiliations:** Department of Mathematics, Çukurova University, 01330 Adana, Turkey

**Keywords:** Primitive element, Free Lie algebra, Ideal, Lower central series, Free nilpotent

## Abstract

Let *F* be a free Lie algebra of finite rank over a field *K*. We prove that if an ideal $\left\langle \tilde{v}\right\rangle$ of the algebra $F/\gamma _{m+1}\left( F^{\prime }\right)$ contains a primitive element $\tilde{u}$ then the element $\tilde{v}$ is primitive. We also show that, in the Lie algebra $F/\gamma _{3}\left( F\right) ^{\prime }$ there exists an element $\bar{v}$ such that the ideal $\left\langle \bar{v}\right\rangle$ contains a primitive element $\bar{u}$ but, $\bar{u}$ and $\bar{v}$ are not conjugate by means of an inner automorphism.

## Background

Let *F* be a free Lie algebra of finite rank $n$, with $n\ge 2$, freely generated by the set $\left\{ x_{1},\ldots ,x_{n}\right\}$ over a field *K*. By $F^{\prime }$ and $F^{\prime \prime }$ we denote the subalgebras $\left[ F,F\right]$ and $\left[ F^{\prime },F^{\prime }\right]$ of *F* respectively. An ideal *V* in the free Lie algebra *F* is called a verbal ideal if for any $g(x_{1},\ldots ,x_{n})\in V$ and any $h_{1},\ldots ,h_{n}\in F$ the Lie polynomial $g(h_{1},\ldots ,h_{n})$ belongs to *V*. Let *V* be a non-trivial verbal ideal of *F*. An element of *F* is said to be primitive if it can be included in a free generating set of *F*. Similarly an element of the relatively free Lie algebra *F* / *V* is called primitive if it is extendible to a free generating set of *F* / *V*.

Let $L=F/ F^{\prime \prime }$ be the free metabelian Lie algebra. Write $\overline{x}_{i}=x_{i}+F^{\prime \prime },i=1,2,\ldots ,n.$ Thus, the set $\left\{ \overline{x}_{1},\ldots ,\overline{x}_{n}\right\}$ is a free generating set for *L* (Bahturin 1987). For $g\in L$, let $\left\langle g\right\rangle$ be the ideal generated by *g* and let *h* be a primitive element of *L*. It is known that if $h\in \left\langle g\right\rangle$ then *g* is a primitive element in *L* (Chirkov and Shevelin 2001). In fact there is an inner automorphism $\theta$ of *L* such that $\theta \left( h\right) =g$. For each $\ v\in L^{\prime }$ the linear operator

$$\begin{aligned} adv:L\longrightarrow L \end{aligned}$$

defined by

$$ adv\left( w\right) =\left[ w,v\right] ,\quad w\in L $$

is a derivation of *L* and $ad^{2}v=0$ because $L^{\prime \prime }=\left\{ 0\right\}$. Hence the linear mapping

$$\begin{aligned} \exp \left( adv\right) =1+adv \end{aligned}$$

is well defined and it is an inner automorphism of *L*. In Chirkov and Shevelin (2001) proved that for $g\in L$ if a primitive element *h* of $\ L$ belongs to the ideal $\left\langle g\right\rangle$ then *h* and *g* are conjugate by means of an inner automorphism of *L*. This result was obtained by Evans (1994) for free metabelian groups. Does a similar result, as in *L*, holds for the Lie algebras $F/ \gamma _{m+1}\left( F^{\prime }\right)$ and $F/ \gamma _{3}\left( F\right) ^{\prime }$? In the group case this question was answered by Timoshenko (1997). In the present paper we answer this question. We obtain an affirmative answer for the Lie algebra $F/ \gamma _{m+1}\left( F^{\prime }\right)$. In contrast to the case of free metabelian Lie algebras and free Lie algebras of the form $F/ \gamma _{m+1}\left( F^{\prime }\right)$, for the Lie algebra $F/ \gamma _{3}\left( F\right) ^{\prime }$ we prove that the question has a negative answer. Our main results are similar to the result of Timoshenko (1997) in the case of groups but there are some essential differences.

## Preliminaries

Let *F* be the free Lie algebra generated by a set $$X=\left\{ x_{1},\ldots ,x_{n}\right\} $$ over a field *K* of characterisitic zero$$, U\left( F\right) $$ be the universal enveloping algebra of *F* and $$\Delta $$ its augmentation ideal, that is, the kernel of the natural homomorphism $$ \sigma :U\left( F\right) \longrightarrow K$$ defined by $$\sigma \left( x_{i}\right) =0,1\le i\le n$$. For a given subalgebra *R* of *F* we denote by $$\Delta _{R}$$ the left ideal of $$U\left( F\right) $$ generated by the subalgebra *R*. In the case where *R* is an ideal of *F*, $$\Delta _{R}$$ becomes a two-sided ideal of $$U\left( F\right) .$$ In fact $$\Delta _{R}$$ is the kernel of the natural homomorphism $$U\left( F\right) \longrightarrow $$$$ U\left( F/ R\right) $$. For any element *u* of *F* we denote by $$ \left\langle u\right\rangle $$ the ideal of *F* generated by the element *u*.

Fox (1953) gave a detailed account of the differential calculus in a free group ring. We introduce here free derivations $\frac{\partial }{ \partial x_{i}}:U\left( F\right) \longrightarrow U\left( F\right) ,1\le i\le n$ such that $\frac{\partial }{\partial x_{i}}\left( x_{j}\right) =\delta _{ij}\left( \text {Kronecker delta}\right) ,\frac{\partial \left( uv\right) }{\partial x_{i}}=\frac{\partial u}{\partial x_{i}}\sigma \left( v\right) +u\frac{\partial v}{\partial x_{i}}$. It is an obvious consequence of the definitions that $\frac{\partial }{\partial x_{i}}\left( 1\right) =0$ . The ideal $\Delta$ is a free left $U\left( F\right)$-module with a free basis *X* and the mappings $\frac{\partial }{\partial x_{i}}$ are projections to the corresponding free cyclic direct summands. Thus any element $f\in \Delta$ can be uniquely written in the form

$$\begin{aligned} f=\sum \limits _{i=1}^{n}\frac{\partial f}{\partial x_{i}}x_{i}. \end{aligned}$$

For any elements $g_{1},\ldots ,g_{n}$ of $U\left( F\right)$ we can always find an element *f* of $U\left( F\right)$ such that $\frac{\partial f}{\partial x_{i}}=g_{i},1\le i\le n.$

Let $\partial f$ be the column vector $\left( \frac{\partial f}{\partial x_{1}},\ldots ,\frac{\partial f}{\partial x_{n}}\right) ^{T}$, where *T* indicates transpose.

For any Lie algebra *G*,  the lower central series

$$\begin{aligned} G=\gamma _{1}(G)\supseteq \gamma _{2}(G)\supseteq \cdots \supseteq \gamma _{k}(G) \supseteq \cdots \end{aligned}$$

is defined inductively by $\gamma _{2}(G)=[G,G], \gamma _{k}(G)=[\gamma _{k-1}(G),G], k\geqslant 2.$ We usually write $G^{\prime },$ for $\gamma _{2}\left( G\right) .$

Let *R* be an ideal of *F*. If *u* is an element of *F*, then we denote the images of *u* under the natural homomorphisms as follows: by $\widehat{u}$ in *F* / *R*,  by $\overline{u}$ in $F/ R^{\prime }$ and by $\widetilde{u}$ in $F/ \gamma _{m+1}\left( R\right) ,$ where $m\ge 1.$

In (Umirbaev 1993), Umirbaev has defined the right derivatives in the algebras $F/ R^{\prime }$ and $F/ \gamma _{m+1}\left( R\right) .$ We give a summary here referring to (Umirbaev 1993).

Let

$$\begin{aligned} \rho :\left[ U\left( F\right) ^{n}\right] ^{T}\rightarrow \left[ U\left( F/ R\right) ^{n}\right] ^{T}, \end{aligned}$$

be the natural componentwise homomorphism, i.e.,

$$\begin{aligned} \rho \left( \left( f_{1},\ldots ,f_{n}\right) ^{T}\right) =\left( \widehat{f_{1}} ,\ldots ,\widehat{f_{n}}\right) ^{T}. \end{aligned}$$

where $\left( \widehat{f_{1}},\ldots ,\widehat{f_{n}}\right) ^{T}$ is the transpose of the vector $\left( \widehat{f_{1}},\ldots ,\widehat{f_{n}}\right) .$

Consider the composition mapping

1 $$\begin{aligned} \rho \circ \partial :F\overset{\partial }{\rightarrow }\left[ U\left( F\right) ^{n}\right] ^{T}\overset{\rho }{\rightarrow }\left[ U\left( F/ R\right) ^{n}\right] ^{T}. \end{aligned}$$

This mapping induces the mappings

$$\begin{aligned} \overline{\partial }:F/ R^{\prime }\rightarrow \left[ U\left( F/ R\right) ^{n}\right] ^{T}, \quad \widetilde{\partial }:F/ \gamma _{m+1}\left( R\right) \rightarrow \left[ U\left( F/ R\right) ^{n}\right] ^{T}. \end{aligned}$$

Since the kernel of the mapping $\widetilde{\partial }$ is $R^{\prime }/ \gamma _{m+1}\left( R\right)$ (see Umirbaev 1993 for details) then it induces the mapping $\overline{\overline{ \partial }}:H\rightarrow \left[ U\left( F/ R\right) ^{n}\right] ^{T},$ where $H=F/ \gamma _{m+1}\left( R\right) / R^{\prime }/ \gamma _{m+1}\left( R\right)$.

For any element f of *F* the components $$\frac{\overline{\partial }\ \overline{f}}{\partial _{x_{i}}},$$$$\frac{\widetilde{\partial }\ \widetilde{f}}{\partial _{x_{i}}}$$ and $$\frac{\overline{\overline{\partial }}\ \overline{\overline{f}}}{\partial _{x_{i}}}$$ of the vectors

$$\begin{aligned} \overline{\partial }\left( \overline{f}\right) =\left( \frac{\overline{ \partial }\ \overline{f}}{\partial _{x_{1}}},\ldots ,\frac{\overline{ \partial }\ \overline{f}}{\partial _{x_{n}}}\right) ^{T},\ \widetilde{\partial }\left( \widetilde{f}\right) =\left( \frac{\widetilde{ \partial }\widetilde{f}}{\partial _{x_{1}}},\ldots ,\frac{\widetilde{\partial } \widetilde{f}}{\partial _{x_{n}}}\right) ^{T},\ \overline{\overline{ \partial }}=\left( \frac{\overline{\overline{\partial }}\ \overline{ \overline{f}}}{\partial _{x_{1}}},\ldots ,\frac{\overline{\overline{\partial }} \ \overline{\overline{f}}}{\partial _{x_{n}}}\right) \end{aligned}$$

are called the partial derivatives of $$\overline{f},$$$$\widetilde{f}$$ and $$\overline{\overline{f}}$$ respectively. Here we use left derivatives instead of right derivatives.

For each $$u\in R/ \gamma _{m+1}\left( R\right) $$ the derivation $$ adu:\ F/ \gamma _{m+1}\left( R\right) \longrightarrow $$$$F/ \gamma _{m+1}\left( R\right) $$ is nilpotent and $$\left( adu\right) =0,$$ because $$\gamma _{m+1}\left( \ R/ \gamma _{m+1}\left( R\right) \right) =\left\{ 0\right\} .$$

Hence the linear mapping

$$\begin{aligned} \exp \left( adu\right) =1+\frac{adu}{1!}+\frac{ad^{2}u}{2!}+\cdots +\frac{ad^{m}u }{m!} \end{aligned}$$

is well defined and it is an inner automorphism of $F/ \gamma _{m+1}\left( R\right) ,m\ge 1,$ that is, since $[[w,\underset{\left( m+1\right) -times}{\underbrace{u],\ldots ,u}}]=0,$

$$\begin{aligned} \exp \left( adv\right) \left( w\right) =w+\frac{\left[ w,u\right] }{1!}+ \frac{\left[ \left[ w,u\right] ,u\right] }{2!}+\cdots +\frac{[[w,\overset{ m-times }{\overbrace{u],\ldots ,u}}]}{m!}. \end{aligned}$$

We need the following technical lemmas. The first lemma is an immediate consequence of the definitions.

### **Lemma 1**

*LetJ be an arbitrary ideal of*$U\left( F\right)$* and*$u\in \Delta$.* Then*$u\in J\Delta$* if and only if*$\frac{\partial u}{\partial x_{i}} \in J$* for each*$i,1\le i\le n.$

The next lemma can be found in Yunus (1984).

### **Lemma 2**

*LetR be an ideal ofF and*$u\in F$.* Then*$u\in \Delta _{R}\Delta$* if and only if*$u\in R^{\prime }$.

## Main results

Let *F* be the free Lie algebra generated by a set $X=\left\{ x_{1},\ldots ,x_{n}\right\} ,n\ge 2,$ over a field *K* of characteristic zero and let *R* be a non-trivial verbal ideal of *F*.

For an element *f* of *F* the vector $\left( \frac{\partial f}{\partial x_{1} },\ldots ,\frac{\partial f}{\partial x_{n}}\right)$ is called unimodular, if there exist $a_{1},\ldots ,a_{n}\in U\left( F\right)$ such that

$$\begin{aligned} a_{1}\frac{\partial f}{\partial x_{1}}+\cdots +a_{n}\frac{\partial f}{\partial x_{n}}=1. \end{aligned}$$

Umirbaev (1993) has proved a criterion of primitiveness for a system of elements in a finitely generated free Lie algebra of the form $F/ \gamma _{m+1}\left( R\right)$, where $m\ge 1$ and $R=F^{\prime }$. Umirbaev’s criterion for the primitivity of an element of the algebra $F/ \gamma _{m+1}\left( R\right)$ is stated below.

### **Proposition 3**

*Let*$\ R=F^{\prime }$.* An element*$\widetilde{u}$* of*$F/ \gamma _{m+1}\left( R\right)$* is primitive if and only if the vector*$\left( \frac{ \widetilde{\partial }\widetilde{u}}{\partial x_{1}},\ldots ,\frac{\widetilde{ \partial }\widetilde{u}}{\partial x_{n}}\right)$* is unimodular in*$U\left( F/ R\right)$.

We are going to consider the case $R=F^{\prime }$.

### **Proposition 4**

*An element*$\overline{f}$* of the free metabelian Lie algebra*$F/ F^{\prime \prime }$* is primitive if and only if the image*$\widetilde{f}$* is primitive in the free nilpotent-by-abelian Lie algebra*$F/ \gamma _{m+1}\left( F^{\prime }\right) ,$* where*$f\in F,\ m\ge 2$.

### Proof

Suppose that the element $$\overline{f}$$ of $$F/ F^{\prime \prime }$$ is primitive. If we put $$m=1$$ in Proposition 3 we have that the vector $$ \left( \frac{\overline{\partial }\ \overline{f}}{\partial x_{1}},\ldots , \frac{\overline{\partial }\ \overline{f}}{\partial x_{n}}\right) $$ is unimodular in $$U\left( F/ F^{\prime }\right) ,$$ that is, there exist $$ a_{1}$$,…,$$a_{n}\in U\left( F/ F^{\prime }\right) $$ such that $$ \sum \nolimits _{i=1}^{n}a_{i}\frac{\overline{\partial }\ \overline{f}}{ \partial x_{i}}=1$$.

Let $H=F/ \gamma _{m+1}\left( F^{\prime }\right) / F^{\prime \prime }/ \gamma _{m+1}\left( F^{\prime }\right)$. We calculate the derivative $\frac{\overline{\partial }\ \overline{f}}{\partial x_{i}}$ by using the natural homomorphism $\theta :F/ \gamma _{m+1}\left( F^{\prime }\right) \rightarrow H,$ the isomorphism $\varphi :H\rightarrow F/ F^{\prime \prime }$ and the chain rule for derivatives:

$$\begin{aligned} \frac{\overline{\partial }\ \overline{f}}{\partial x_{i}} &=  \frac{ \overline{\partial }\varphi \left( \overline{\overline{f }}\right) }{\partial x_{i}} \\ &= \frac{\overline{\partial }\varphi \left( \overline{\overline{f}}\right) }{ \partial \overline{\overline{f}}}.\frac{\overline{\overline{\partial }}\ \overline{\overline{f}}}{\partial x_{i}} \\ &= \frac{\overline{\partial }\varphi \left( \overline{\overline{f}}\right) }{ \partial \overline{\overline{f}}}.\frac{\overline{\overline{\partial }} \theta \left( \widetilde{f}\right) }{\partial x_{i}} \\&= \frac{\overline{\partial }\varphi \left( \overline{\overline{f}}\right) }{ \partial \overline{\overline{f}}}.\frac{\overline{\overline{\partial }} \theta \left( \widetilde{f}\right) }{\partial \widetilde{f}}.\frac{ \widetilde{\partial }\ \widetilde{f}}{\partial x_{i}}. \end{aligned}$$

It is clear that $\frac{\overline{\partial }\varphi \left( \overline{ \overline{f}}\right) }{\partial \overline{\overline{f}}},\frac{\overline{ \overline{\partial }}\theta \left( \widetilde{f}\right) }{\partial \widetilde{f}}\in U\left( F/ F^{\prime }\right) .$ Therefore from the equality $\sum \nolimits _{i=1}^{n}a_{i}\frac{\overline{\partial }\ \overline{f}}{\partial x_{i}}=1$ we get $\sum \nolimits _{i=1}^{n}b_{i}\frac{ \widetilde{\partial }\ \widetilde{f}}{\partial x_{i}}=1,$ where $b_{i}=a_{i}\frac{\overline{\partial }\varphi \left( \overline{\overline{f}} \right) }{\partial \overline{\overline{f}}}.\frac{\overline{\overline{ \partial }}\theta \left( \widetilde{f}\right) }{\partial \widetilde{f}}.$ Hence by Proposition 3, $\widetilde{f}$ is primitive in $F/ \gamma _{m+1}\left( F^{\prime }\right)$.

Now suppose that $\widetilde{f}$ is primitive element of the algebra $F/ \gamma _{m+1}\left( F^{\prime }\right) .$ By definition it can be extended to a free generating set $Y=\left\{ \widetilde{f}=\widetilde{f} _{1},\ldots ,\widetilde{f}_{n}\right\}$ of $F/ \gamma _{m+1}\left( F^{\prime }\right) .$ Clearly *Y* is linearly independent modulo $\left( F/ \gamma _{m+1}\left( F^{\prime }\right) \right) ^{\prime }$. Therefore the image $\theta \left( Y\right)$ of *Y* in the algebra *H* is linearly independent modulo $H^{\prime }$. As a simple application of theorem 4.2.4.9 of Bahturin (1987) we see that $\theta \left( Y\right)$ freely generates the algebra *H*. Hence the image of $\theta \left( Y\right)$ under the isomorphism $\varphi :H\rightarrow F/ F^{\prime \prime }$ generates the algebra $F/ F^{\prime \prime }.$ That is, the algebra $F/ F^{\prime \prime }$ is freely generated by the set $\varphi \left( \theta \left( Y\right) \right) =\left\{ \overline{f}=\overline{f}_{1},\ldots , \overline{f}_{n}\right\} .$ Thus, $\overline{f}$ is a primitive element of the algebra $F/ F^{\prime \prime }$. $\square$

As a consequence of the result of Chirkov and Shevelin (2001), we obtain the following proposition. Although its proof is given in Ersalan and Esmerligil (2014), our proof is more explicit. The idea of the proof is similar to the idea of the proof of Proposition 2 of the paper by Timoshenko (1997) for groups.

### **Proposition 5**

*Let*$\widetilde{u}$* be a primitive element of the algebra*$F/ \gamma _{m+1}\left( F^{\prime }\right)$* and let*$\widetilde{v}\in F/ \gamma _{m+1}\left( F^{\prime }\right)$* where*$u,v\in F,m\ge 1$. * If*$\widetilde{u}\in \left\langle \widetilde{v}\right\rangle$* then*$\widetilde{ v }$* is also primitive*.

### Proof

Let $$\widetilde{u},\widetilde{v}$$$$\in F/ \gamma _{m+1}\left( F^{\prime }\right) .$$ Assume that $$\widetilde{u}$$ is primitive and that it is contained in the ideal $$\left\langle \widetilde{v}\right\rangle $$ of $$ F/ \gamma _{m+1}\left( F^{\prime }\right) $$. By Proposition 4, $$ \overline{u}$$ is primitive. In the view of Proposition 4, it sufficies to the prove that the element $$\overline{v}$$ of $$F/ F^{\prime \prime }$$ is primitive.

Since

$$\begin{aligned} \widetilde{u}=u+\gamma _{m+1}\left( F^{\prime }\right) \in \left\langle v+\gamma _{m+1}\left( F^{\prime }\right) \right\rangle ,\quad m\ge 1 \end{aligned}$$

we have

$$\begin{aligned} u\in \left\langle v\right\rangle \left( mod F^{\prime \prime }\right) , \end{aligned}$$

that is,

$$\begin{aligned} \overline{u}=u+F^{\prime \prime }\in \left\langle v+F^{\prime \prime }\right\rangle . \end{aligned}$$

From the result of Chirkov and Shevelin (2001), we obtain that the elements $\overline{u}$ and $\overline{v}$ are conjugate by means of an inner automorphism. Therefore $\overline{v}$ is primitive. Hence the result follows. $\square$

The mapping $\ \widehat{}:F\rightarrow F/ R$ can be extended to the mapping $\ \widehat{}:U\left( F\right) \rightarrow U\left( F/ R\right)$ for which we preserve the same notation.

The following lemma will play a crucial role in proving our main result.

### **Lemma 6**

*LetR be a verbal ideal of*$F,r\in R$* and let*$v\in F.$* Then*$r+R^{\prime }\in \left\langle v\right\rangle +R^{\prime }$* if and only if there exist an element*$\widehat{\alpha }\in U\left( F/R\right)$* and an element*$\widehat{\beta _{i}}\in \Delta _{\widehat{v}},$* such that*$\frac{\widehat{\partial r}}{\partial x_{i}}=\widehat{\alpha }\frac{ \widehat{\partial v}}{\partial x_{i}}+\widehat{\beta _{i}},$*where *$i=1,\ldots ,n$* and*$\Delta _{\widehat{v}}$* is the ideal generated by the element*$\widehat{v}$* in the algebra*$U\left( F/R\right) .$

### Proof

Let *r* be an element of the ideal *R*, $v\in F$ and $\overline{r}\in \left\langle \overline{v}\right\rangle .$ Then $r\in \left\langle v\right\rangle \left( modR^{\prime }\right) ,$ where $\left\langle v\right\rangle$ is the ideal of *F* generated by *v*. Any element of the ideal $\left\langle v\right\rangle$ can be written as linear combinations of commutators of *F* depending on the element *v*. Applying the Jacobi identitiy and the anticommutativity, these commutators can be rewritten as linear combinations of commutators of the form

2 $$\begin{aligned} \left[ \left[ \ldots \left[ \left[ v,x_{i_{1}}\right] ,x_{i_{2}}\right] ,\ldots \right] ,x_{i_{k}}\right] ,\quad x_{i_{1}},\ldots x_{i_{k}}\in \left\{ x_{1},\ldots ,x_{n}\right\} ,\quad k\ge 0. \end{aligned}$$

If $r\equiv v\left( modR^{\prime }\right)$ then clearly $\frac{ \widehat{ \partial r}}{\partial x_{i}}=\frac{\widehat{\partial v}}{\partial x_{i}} ,i=1,\ldots n.$

Now assume that the element *r* is written as a linear combination of elements of the form (2). Without loss of generality we may assume that

$$\begin{aligned} r\equiv \left[ \left[ \ldots \left[ v,x_{i_{1}}\right] ,\ldots \right] ,x_{i_{k}} \right] \left( modR^{\prime }\right) ,\quad k\ge 1, \end{aligned}$$

By straightforward calculations we see that the form of the derivatives $\frac{\partial r}{\partial x_{i}}$ are

$$\begin{aligned} \frac{\partial r}{\partial x_{i}}=\alpha \frac{\partial v}{\partial x_{i}} +\beta _{i}+\Delta _{R}, \end{aligned}$$

where $$\alpha \in U\left( F/ R\right) ,\beta _{i}\in \Delta _{v},i=1,\ldots, n.$$

Therefore

$$\begin{aligned} \frac{\widehat{\partial r}}{\partial x_{i}}=\widehat{\alpha }\frac{\widehat{ \partial v}}{\partial x_{i}}+\widehat{\beta _{i}}. \end{aligned}$$

Let now $\frac{\widehat{\partial r}}{\partial x_{i}}=\widehat{\alpha } \frac{\widehat{\partial v}}{\partial x_{i}}+\widehat{\beta _{i}},$ where $\widehat{\alpha }\in U\left( F/R\right) ,\widehat{\beta _{i}}\in \Delta _{ \widehat{v}},i=1,\ldots n$.

The kernel of the natural homomorphism $\widehat{}:U\left( F\right) \longrightarrow U\left( F/R\right)$ is $\Delta _{R},$ and hence

$$\begin{aligned} \frac{\partial r}{\partial x_{i}}+\Delta _{R}=\alpha \frac{\partial v}{ \partial x_{i}}+\beta _{i}+\Delta _{R}. \end{aligned}$$

Then there exists an element *g* of $$\Delta _{R}$$ such $$\frac{\partial }{ \partial x_{i}}\left( r-\alpha v\right) =\beta _{i}+g,$$ where $$\beta _{i}\in \Delta _{v},$$ that is, $$\frac{\partial }{\partial x_{i}} \left( r-\alpha v\right) \in \Delta _{v}+\Delta _{R}.$$ By Lemma 1 we have $$ r-\alpha v\in \Delta _{v}\Delta +\Delta _{R}\Delta .$$ Hence the element $$r-\alpha v$$ of *F* can be written as $$r-\alpha v=h+z,$$ where $$h\in \Delta _{v}\Delta ,$$$$z\in \Delta _{R}\Delta .$$ By Lemma 2 we get $$h\in \left\langle v\right\rangle ^{\prime }$$ and $$\ z\in R^{\prime }.$$ Hence $$r+R^{\prime }=\alpha v+h+R^{\prime }.$$ This completes the proof. $$\square $$

In contrast to the case of free metabelian Lie algebras we can show that there exists an element $\overline{v}$ of the algebra $F/ \gamma _{3}\left( F\right) ^{\prime }$ such that the ideal $\left\langle \overline{v }\right\rangle$ of $F/ \gamma _{3}\left( F\right) ^{\prime }$ contains a primitive element $\overline{u},$ but $\overline{u}$ and $\overline{v}$ are not conjugate by means of an inner automorphism.

### **Theorem 7**

*There is an element*$\overline{v}$* in the algebra*$F/ \gamma _{3}\left( F\right) ^{\prime }$* such that the ideal*$\left\langle \overline{ v }\right\rangle$* of*$F/ \gamma _{3}\left( F\right) ^{\prime }$* contains the element*$\overline{x_{1}}$ ,* but the elementsv and*$x_{1}$* are not conjugate modulo*$\gamma _{3}\left( F\right) ^{\prime }$* by means of an inner automorphism.*

### Proof

We consider the element $\overline{v}=x_{1}+\left[ \left[ \left[ \left[ x_{1},x_{2}\right] ,x_{2}\right] ,x_{1}\right] ,x_{2}\right] +\gamma _{3}\left( F\right) ^{\prime }$ of $F/ \gamma _{3}\left( F\right) ^{\prime }$ which is an analogue of the element given in Fox (1953) for groups. Let $w=\left[ \left[ \left[ \left[ x_{1},x_{2}\right] ,x_{2}\right] ,x_{1}\right] ,x_{2}\right] .$

We have

$$\begin{aligned} \frac{\partial w}{\partial x_{1}} &=  -x_{2}\cdot\left[ \left[ x_{1},x_{2}\right] ,x_{2}\right] +x_{2}\cdot x_{1}\cdot\frac{\partial \left[ \left[ x_{1},x_{2}\right] ,x_{2}\right] }{\partial x_{1}}, \\ \frac{\partial w}{\partial x_{2}}&= \left[ \left[ \left[ x_{1},x_{2}\right] ,x_{2}\right] ,x_{1}\right] +x_{2}\cdot x_{1}\cdot\frac{\partial \left[ \left[ x_{1},x_{2}\right] ,x_{2}\right] }{\partial x_{2}}. \end{aligned}$$

Now consider the images $\frac{\widehat{\partial w}}{\partial x_{i}}$ under the homomorphism

$$\begin{aligned} \widehat{}:U\left( F\right) \longrightarrow U\left( F/\gamma _{3}\left( F\right) \right) ,\quad i=1,2. \end{aligned}$$

Then

$$\begin{aligned} \frac{\widehat{\partial w}}{\partial x_{1}}&= \widehat{x_{2}}\cdot\widehat{ x_{1} }\cdot\frac{\widehat{\partial \left[ \left[ x_{1},x_{2}\right] ,x_{2} \right] }}{ \partial x_{1}}, \\ \frac{\widehat{\partial w}}{\partial x_{2}}&= \widehat{x_{2}}\cdot\widehat{ x_{1} }\cdot\frac{\widehat{\partial \left[ \left[ x_{1},x_{2}\right] ,x_{2} \right] }}{ \partial x_{2}}, \\ \frac{\widehat{\partial w}}{\partial x_{k}}&= 0,\quad k>2. \end{aligned}$$

Clearly $\frac{\widehat{\partial w}}{\partial x_{i}}\in \Delta _{\widehat{ x_{1}}}=\Delta _{\widehat{v}}.$ In the above equalities if we set $\widehat{ \alpha }=0$ and $\widehat{\beta _{i}}=\widehat{x_{2}}\cdot\widehat{x_{1}}\cdot \frac{\widehat{\partial \left[ \left[ x_{1},x_{2}\right] ,x_{2}\right] }}{ \partial x_{i}},i=1,2,$ then we see that

$$\begin{aligned} \frac{\widehat{\partial w}}{\partial x_{i}}=\widehat{\alpha }\cdot\frac{\widehat{ \partial v}}{\partial x_{i}}+\ \widehat{\beta _{i}},\quad i=1,2. \end{aligned}$$

By Lemma 6 $$w+\gamma _{3}\left( F\right) ^{\prime }\in \left\langle v\right\rangle $$$$+\gamma _{3}\left( F\right) ^{\prime }.$$ Therefore we have

$$\begin{aligned} x_{1}+\gamma _{3}\left( F\right) ^{\prime }=v-w+\gamma _{3}\left( F\right) ^{\prime }\in \left\langle v\right\rangle +\gamma _{3}\left( F\right) ^{\prime }. \end{aligned}$$

Now we are going to verify that the element *w* can not be written in the form $\left[ x_{1},u\right]$ in the algebra $F/ \gamma _{3}\left( F\right) ^{\prime }.$

Assume that the rank of *F* equal to $2, u\in \gamma _{3}\left( F\right)$ and

3 $$\begin{aligned} w=\left[ x_{1},u\right] . \end{aligned}$$

Let us calculate the derivative $\frac{\partial }{\partial x_{1}}$ of both sides of (3). We have

$$\begin{aligned} -x_{2}\cdot\left[ \left[ x_{1},x_{2}\right] ,x_{2}\right] +x_{2}\cdot x_{1}\cdot x_{2}\cdot x_{2}=-u+x_{1}\cdot\frac{\partial u}{\partial x_{1}}. \end{aligned}$$

Taking the image under the homomorphism $\widehat{}:U\left( F\right) \longrightarrow U\left( F/\gamma _{3}\left( F\right) \right)$ we get

4 $$ \widehat{x_{2}}\cdot\widehat{x_{1}}\cdot\widehat{x_{2}}\cdot\widehat{x_{2}}=\widehat{ x_{1}}\frac{\widehat{\partial u}}{\partial x_{1}} . $$

It is well known that the set $\left\{ \widehat{x_{1}},\widehat{x_{2}},\left[ \widehat{x_{1}},\widehat{x_{2}}\right] \right\}$ is a basis of $F/\gamma _{3}\left( F\right) .$ Therefore by Poincare–Birkhoff–Witt’s theorem the algebra $U\left( F/\gamma _{3}\left( F\right) \right)$ is a free K- module generated 1 and the all ordered monomials of the form

$$\begin{aligned} \left[ \widehat{x_{1}},\widehat{x_{2}}\right] ^{r}\cdot\widehat{x_{1}}^{s}\cdot \widehat{x_{2}}^{k},\quad r\ge 0,s\ge 0,k\ge 0,\quad \left( r,s,k\right) \ne \left( 0,0,0\right) . \end{aligned}$$

Thus every element of $\ U\left( F/\gamma _{3}\left( F\right) \right)$ can be uniquely written as

5 $$\begin{aligned} \underset{r,s,k\ge 0}{\sum \alpha _{rsk}}\left[ \widehat{x_{1}},\widehat{ x_{2}}\right] ^{r}\cdot\widehat{x_{1}}^{s}\cdot\widehat{x_{2}}^{k},\quad \alpha _{ijk}\in K. \end{aligned}$$

Let us express each side of (4) in the form (5):

$$\begin{aligned} \widehat{x_{2}}\cdot\widehat{x_{1}}\cdot\widehat{x_{2}}\cdot\widehat{x_{2}} &=  \left[ \widehat{x_{2}},\widehat{x_{1}}\right] \widehat{x_{2}}\cdot\widehat{x_{2}}+ \widehat{x_{1}}\cdot\widehat{x_{2}}\cdot\widehat{x_{2}}\cdot\widehat{x_{2}}, \\ \widehat{x_{1}}\frac{\widehat{\partial u}}{\partial x_{1}} & = \widehat{x_{1}} \underset{i,j,k\ge 0}{\sum \alpha _{ijk}}\left[ \widehat{x_{1}},\widehat{ x_{2}}\right] ^{r}\cdot\widehat{x_{1}}^{s}\cdot\widehat{x_{2}}^{k}. \end{aligned}$$

Then

6 $$\begin{aligned} \widehat{x_{1}}\cdot\underset{i,j,k\ge 0}{\sum \alpha _{ijk}}\left[ \widehat{ x_{1}},\widehat{x_{2}}\right] ^{i}\cdot\widehat{x_{1}}^{j}\cdot\widehat{x_{2}}^{k}= \left[ \widehat{x_{2}},\widehat{x_{1}}\right] \cdot\widehat{x_{2}}^{2}+\widehat{ x_{1}}\cdot\widehat{x_{2}}^{3}. \end{aligned}$$

We note that in the algebra $$F/\gamma _{3}\left( F\right) $$ we have $$\left[ \left[ \widehat{x_{1}},\widehat{x_{2}}\right] ,\widehat{x_{i}}\right] =0,$$$$i\in \left\{ 1,2\right\} .$$ That is, the elements $$\left[ \widehat{x_{1}}, \widehat{x_{2}}\right] $$ and $$\widehat{x_{i}}$$ commute in the algebra $$ U\left( F/\gamma _{3}\left( F\right) \right) .$$

So from (6) we get

7 $$\begin{aligned} \underset{i,j\ge 0}{\sum }\alpha _{ij2}\left[ \widehat{x_{1}},\widehat{ x_{2}}\right] ^{i}\cdot\widehat{x_{1}}^{j+1}=\left[ \widehat{x_{2}},\widehat{ x_{1}}\right] \end{aligned}$$

and

$$\begin{aligned} \underset{i,j\ge 0}{\sum }\alpha _{ij3}\left[ \widehat{x_{1}},\widehat{ x_{2}}\right] ^{i}\cdot\widehat{x_{1}}^{j+1}=\widehat{x_{1}}. \end{aligned}$$

Using (7) we obtain

$$\begin{aligned} \underset{j\ge 0}{\sum }\alpha _{1j2}\widehat{x_{1}}^{j+1}=-1, \end{aligned}$$

which is impossible. This contradiction proves the theorem. $\square$

## Conclusions

In this work we found a relation between the generator of a one-generated ideal of a relatively free Lie algebra and a primitive element which is contained in this ideal. One can expects to adopt our results for ideals of some relatively free Lie algebras which have more than one generator.
